# Antitumor Reactive T-Cell Responses Are Enhanced In Vivo by DAMP Prothymosin Alpha and Its C-Terminal Decapeptide

**DOI:** 10.3390/cancers11111764

**Published:** 2019-11-09

**Authors:** Anastasios I. Birmpilis, Chrysoula-Evangelia Karachaliou, Pinelopi Samara, Kyriaki Ioannou, Platon Selemenakis, Ioannis V. Kostopoulos, Nadia Kavrochorianou, Hubert Kalbacher, Evangelia Livaniou, Sylva Haralambous, Athanasios Kotsinas, Farzin Farzaneh, Ioannis P. Trougakos, Wolfgang Voelter, Meletios-Athanasios Dimopoulos, Aristotelis Bamias, Ourania Tsitsilonis

**Affiliations:** 1 Department of Biology, National and Kapodistrian University of Athens, 15784 Athens, Greece; abirmpilis@biol.uoa.gr (A.I.B.); psamara@biol.uoa.gr (P.S.); kikioannou@gmail.com (K.I.); gikosto@gmail.com (I.V.K.); itrougakos@biol.uoa.gr (I.P.T.); 2 Institute of Nuclear and Radiological Sciences and Technology, Energy and Safety, NCSR “Demokritos”, Agia Paraskevi, 15310 Athens, Greece; xrisak15@hotmail.com (C.-E.K.); livanlts@rrp.demokritos.gr (E.L.); 3 King’s College London, Rayne Institute, 123 Coldharbour Lane, SE5 9NU London, UK; farzin.farzaneh@kcl.ac.uk; 4 Molecular Carcinogenesis Group, Department of Histology and Embryology, School of Medicine, National and Kapodistrian University of Athens, 75 Mikras Asias Str, 11527 Athens, Greece; pselemenakis@gmail.com (P.S.); akotsin@med.uoa.gr (A.K.); 5 Inflammation Research Group, Transgenic Technology Laboratory, Hellenic Pasteur Institute, 127 Vasilissis Sofias Avenue, 11521 Athens, Greece; nadia.kavrohorianou@gmail.com (N.K.); sharalambous@pasteur.gr (S.H.); 6 Interfaculty Institute of Biochemistry, University of Tübingen, 72076 Tübingen. Germany; kalbacher@uni-tuebingen.de (H.K.); wolfgang.voelter@uni-tuebingen.de (W.V.); 7 Department of Clinical Therapeutics, School of Medicine, National and Kapodistrian University of Athens, 11528 Athens, Greece; mdimop@med.uoa.gr (M.-A.D.); abamias@med.uoa.gr (A.B.)

**Keywords:** adjuvant, antitumor peptide vaccine, biologic response modifier, danger-associated molecular pattern—DAMP, immunoreactive decapeptide, in vivo melanoma model, proinflammatory cytokine, prothymosin α, Th1-type cytokine

## Abstract

Prothymosin α (proTα) and its C-terminal decapeptide proTα(100–109) were shown to pleiotropically enhance innate and adaptive immune responses. Their activities have been broadly studied in vitro, focusing primarily on the restoration of the deficient immunoreactivity of cancer patients’ leukocytes. Previously, we showed that proTα and proTα(100–109) act as danger-associated molecular patterns (DAMPs), ligate Toll-like receptor-4, signal through TRIF- and MyD88-dependent pathways, promote the maturation of dendritic cells and elicit T-helper type 1 (Th1) immune responses in vitro, leading to the optimal priming of tumor antigen-reactive T-cell functions. Herein, we assessed their activity in a preclinical melanoma model. Immunocompetent mice bearing B16.F1 tumors were treated with two cycles of proTα or proTα(100–109) together with a B16.F1-derived peptide vaccine. Coadministration of proTα or proTα(100–109) and the peptide vaccine suppressed melanoma-cell proliferation, as evidenced by reduced tumor-growth rates. Higher melanoma infiltration by CD3+ T cells was observed, whereas ex vivo analysis of mouse total spleen cells verified the in vivo induction of melanoma-reactive cytotoxic responses. Additionally, increased levels of proinflammatory and Th1-type cytokines were detected in mouse serum. We propose that, in the presence of tumor antigens, DAMPs proTα and proTα(100–109) induce Th1-biased immune responses in vivo. Their adjuvant ability to orchestrate antitumor immunoreactivities can eventually be exploited therapeutically in humans.

## 1. Introduction

Prothymosin alpha (proTα) is a highly acidic polypeptide that was first isolated from rat thymus in 1984 [1]. Although initially regarded as a thymic hormone with exclusive intrathymic function, it was later shown that the polypeptide is ubiquitously expressed in almost all types of mammalian tissue and exerts a dual role; intracellularly, proTα is involved in chromatin remodeling [2], promotes cell proliferation [3], and inhibits apoptosome formation [4], and, extracellularly, proTα acts pleiotropically as a biologic response modifier, regulating the activities of a variety of immune cells [5].

In the context of the latter, proTα was reportedly shown to stimulate the antimicrobial [6] and antiviral properties of macrophages [7], activate monocytes by upregulating human leukocyte antigen (HLA)-DR expression [8,9] and interleukin (IL)-1β production [10], and enhance T-cell proliferation by increasing IL-2 production and IL-2R expression [11,12]. Additionally, both proTα and its C-terminal decapeptidyl fragment proTα(100–109) spanning the nuclear localization signal (NLS) and documented by our team to act as the core immunostimulatory region of proTα [13], were shown in vitro to increase the oxidative and cytotoxic responses of neutrophils [14] and enhance natural-killer (NK) and lymphokine-activated-killer (LAK) cell cytotoxicity synergistically with IL-2 [13]. Furthermore, both molecules induce the in vitro maturation of human monocyte-derived dendritic cells (DCs) [13]. Most importantly, proTα- and proTα(100–109)-matured DCs are also functionally competent to promote Th1-type immune responses in the presence of tumor-associated epitopes via the secretion of soluble mediators like tumor necrosis factor (TNF)-α, interferon (IFN)-γ, and IL-2 [15]. This proinflammatory cytokine profile induced by proTα and proTα(100–109) is likely involved in inflammatory processes, as we recently showed in an in vivo murine model of inflammation, where proTα(100–109), upon systemic administration, selectively accumulated at sites of acute inflammation [16].

Among its multiple extracellular functions, the anticancer properties of proTα comprise the most well-studied area of research. Contrary to the plethora of data that have established the in vitro anticancer activity of proTα, evidence on whether the polypeptide mediates analogous in vivo antitumor responses is limited to only three studies conducted on mice in the 1990s. Specifically, immunocompetent mice inoculated with L1210 leukemic cells and therapeutically treated with proTα survived longer as compared to controls [17]. This likely reflected a cascade of proTα-induced effects that relates to: (i) TNF-α production by peritoneal macrophages, (ii) enhancement of antitumor NK cell cytotoxicity, [18] and (iii) generation of MHC-restricted tumor-specific CD8+ cytotoxic T-cell responses in an IL-2-dependent manner [19]. Thus far, the in vivo anticancer activity of decapeptide proTα(100–109) has not been documented.

Following our previous in vitro study on the anticancer mechanism of action of proTα and proTα(100–109) [15], we assessed the efficacy of both peptides in vivo. A preclinical melanoma model was designed in which mice were treated with proTα or proTα(100–109) in combination with melanoma antigens following a strategy of sequential administration. Tumor-growth rate was monitored, complemented by ex vivo analyses, providing evidence on proTα/proTα(100–109)-induced alterations at the cellular, tissue, and humoral level.

## 2. Results

### 2.1. Therapeutic Administration of ProTα or ProTα(100–109) Suppressed Melanoma Tumor Growth In Vivo

**Figure 1:**
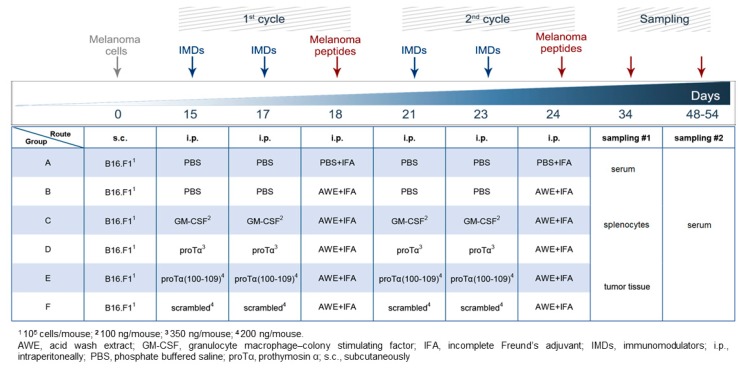
In vivo protocol used to assess effect of proTα and proTα(100–109). Day 0: Mice inoculated subcutaneously (s.c.) with syngeneic B16.F1 melanoma cells. Days 15, 17, 21, and 23: Mice of groups C, D, E, and F administered intraperitoneally (i.p.) granulocyte-macrophage colony-stimulating factor (GM-CSF), proTα, proTα(100–109), or scrambled decapeptide, respectively. Days 18 and 24: Mice of groups B, C, D, E, and F were administered i.p. melanoma peptide extract (AWE) mixed with incomplete Freund’s adjuvant (IFA). Sampling performed on days 34 and 48–54 as indicated.

**Figure 2:**
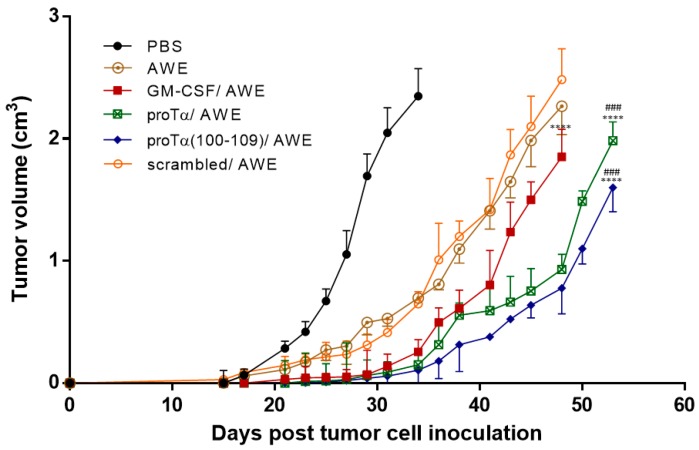
Effect of proTα and proTα(100–109) on melanoma tumors in vivo. Mice were s.c. inoculated with B16.F1 cells (day 0) and, upon palpable tumor formation (day 15), i.p. treated with PBS (control; black curve) or AWE alone (brown), in conjunction with GM-CSF (red), proTα (green), proTα(100–109) (blue), or scrambled peptide (orange curve). For protocol details, see Figure 1. Tumor growth was monitored for up to 54 days. Mean tumor volumes ± SD from 8–10 mice/group are shown. **** *p* < 0.0001 compared to AWE; ^###^ *p* < 0.001 compared to GM-CSF/AWE.

Mice inoculated with syngeneic B16.F1 melanoma cells were treated with two cycles of immunomodulators (IMDs) in combination with a vaccine containing antigenic epitopes eluted from the same melanoma cells (acid wash extract; AWE) (Figure 1). Tumor growth was monitored until tumors reached ~2.5 cm^3^ or until day 54. As shown in Figure 2, tumor volume (in cm^3^) in control mice (administered phosphate-buffered saline; PBS) grew rapidly and, on day 34 post B16.F1 cell inoculation, animals were euthanized for ethical reasons as tumors exceeded 2.5 cm^3^. Animals only treated with the peptide vaccine (AWE) showed reduced tumor-growth rates compared to controls (*p* < 0.01 compared to PBS); this reduction was more evident in mice treated with two cycles of the combination of granulocyte-macrophage colony-stimulating factor (GM-CSF)/AWE (*p* < 0.0001 compared to AWE), while the most significant delay in melanoma tumor growth was recorded in mice therapeutically treated with two cycles of the combined proTα/AWE or proTα(100–109)/AWE preparations. These latter mice retained tumor volumes below 2 cm^3^, even on day 54 (1.9 and 1.6 cm^3^, respectively; *p* < 0.0001 compared to AWE; *p* < 0.001 compared to GM-CSF/AWE). Mice treated with scrambled peptide/AWE showed a tumor-increase rate similar to animals administered only AWE, suggesting that the effect of proTα(100–109) was specific. Altogether, until day 27, i.e., during the two cycles of treatment, the tumors marginally grew in animals receiving the combination of IMD/vaccine and mean tumor volumes in mice administered GM-CSF/AWE, proTα/AWE and proTα(100–109)/AWE were 0.053, 0.030, and 0.023 cm^3^, respectively. On the same day, melanoma tumors in animals treated with AWE or scrambled peptide/AWE showed a ca. 10-fold increase in their masses, 0.304 and 0.236 cm^3^, respectively, whereas control mice developed larger tumors with a mean volume of 1.054 cm^3^. This finding suggests that, in the AWE or scrambled peptide/AWE groups, the B16.F1-extracted peptides were suboptimally presented to immune cells in vivo, whereas the concomitant administration of IMDs and autologous tumor peptide vaccines promoted the activation of highly competent tumor-reactive immune effectors providing a prolonged survival advantage to the animals.

### 2.2. Melanomas from Mice Treated with proTα/AWE or proTα(100–109)/AWE Were Infiltrated by T Cells

**Figure 3:**
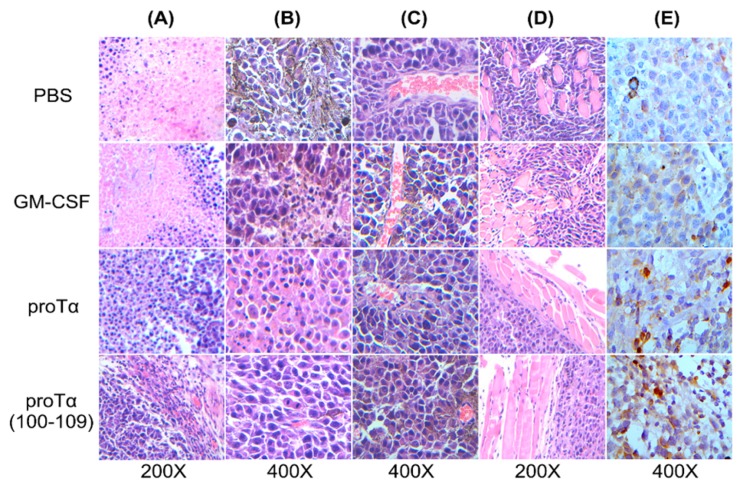
Beneficial modulation of melanoma tumor microenvironment by proTα and proTα(100–109). Sections from paraffin-embedded tumors harvested from mice (*n* = 5/group) on day 34, were stained with hematoxylin-eosin and microscopically examined for the presence of (**A**) necrosis, (**B**) melanin accumulation, (**C**) vascular invasion, and (**D**) smooth muscle invasion. Sections were also stained with an antibody raised against CD3 (**E**); 200× and 400× magnification marked. Representative photographs shown from tumors of mice receiving PBS (control), GM-SCF/AWE, proTα/AWE, and proTα(100–109)/AWE.

Sections from paraffin-embedded melanoma tumors resected from treated animals on day 34 were stained with hematoxylin–eosin and microscopically examined for the presence of necrotic areas, melanin accumulation, vascular, and smooth muscle fiber invasion. As shown in Figure 3, sections from control and GM-CSF/AWE-treated animals had wide necrotic areas where cell fragmentation, membrane disruption, and loss of nuclei resulted in complete loss of tissue architecture; analogous sections from tumors of proTα/AWE- or proTα(100–109)/AWE-treated mice showed significantly less or no necrosis (Figure 3A). Melanin pigmentation was evident in tumors from control mice, less apparent in mice administered GM-CSF/AWE, and almost absent in proTα/AWE- or proTα(100–109)/AWE-treated mouse tumors (Figure 3B), indicating selective elimination of melanoma cells; in support, vascular and muscle invasion by melanoma cells was also highly reduced in mice treated with proTα/AWE or proTα(100–109)/AWE (Figure 3C,D). Most importantly, immunohistochemistry with a CD3 antibody showed very few CD3-positive (CD3+) T cells infiltrating the tumor mass in control mice; noticeable, but still low CD3+ T-cell infiltration in GM-CSF/AWE-treated mice; sections from proTα/AWE- and especially proTα(100–109)/AWE-treated animals were enriched in CD3+ tumor-infiltrating T cells (Figure 3E). These results suggest that the in vivo treatment of melanoma-bearing mice with proTα/AWE or proTα(100–109)/AWE resulted in the selective elimination of melanoma cells and possibly lower metastatic potential. This readout is likely correlated with the formation of a tumor microenvironment permissive for infiltration by activated CD3+ T cells, which accordingly contributed to the aforementioned reduction of tumor mass.

### 2.3. ProTα/AWE and proTα(100–109)/AWE Induced T- and NK Cell-Mediated Cytotoxic Responses In Vivo

**Figure 4:**
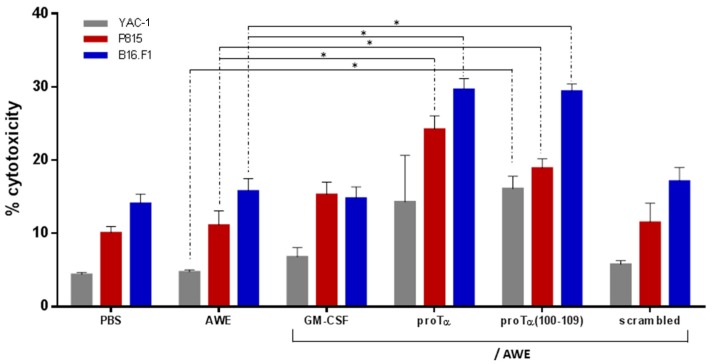
Combined administration of proTα or proTα(100–109) with AWE promoted T- and NK effector cell cytotoxicity in vivo. Cytotoxicity was measured by standard ^51^Cr-release assay. Total spleen cells from treated mice were used as effectors (E) against target (T) YAC-1, P815, and B16.F1 cells at an E:T ratio of 100:1. Mean % cytotoxicity ± SD from five mice/group is shown. * *p* < 0.05 compared to AWE.

To verify whether the in vivo reduction of melanoma tumor growth was associated with the generation of increased antitumor immune responses mediated by melanoma antigen-reactive effectors, selected mice from each group were sacrificed on day 34. Spleens were aseptically removed, and total spleen cells with no additional ex vivo stimulation were immediately used as effectors in standard ^51^Cr-release cytotoxicity assays. Three mouse cell lines were used as targets: (i) YAC-1 cells, sensitive to non-MHC-restricted lysis by NK effector cells; (ii) P815 cells, sensitive to lysis by LAK cells, also in a non-MHC-restricted manner; and (iii) B16.F1 cells that express the melanoma antigens included in AWE and are therefore lysed by MHC-restricted AWE-reactive T-cell effectors. As shown in Figure 4, total spleen cells isolated from proTα/AWE- or proTα(100–109)/AWE-treated mice killed the syngeneic B16.F1 targets (29.7% and 28.9%, respectively) more efficiently compared to cell death induced by the total spleen cells of control (14.1%), AWE-treated (15.8%), GM-CSF/AWE-treated (14.8%), and scrambled peptide/AWE-treated (17.1%) mice (in all cases *p* < 0.05). NK cytotoxicity vs YAC-1 cells was lower in all groups, but higher percentages were observed in proTα/AWE- or proTα(100–109)/AWE-treated mice (14.3% and 16.1%, respectively), compared to 4.4%, 4.8%, 6.8%, and 5.8% in PBS, AWE-, GM-CSF/AWE- and scrambled peptide/AWE-treated animals, respectively. Compared to the control animals (10.1%), LAK cytotoxicity versus P815 targets was marginally increased in AWE- (11.1%) and scrambled peptide/AWE-treated mice (11.5%). LAK cytotoxicity was higher in mice administered GM-CSF/AWE (15.3%) and proTα(100–109)/AWE (18.9%) (*p* < 0.05 compared to AWE), whereas the highest cytotoxicity was recorded in proTα/AWE-treated animals (24.2%; *p* < 0.05 compared to AWE). These results suggest that administration of proTα or proTα(100–109) in conjunction with the peptide vaccine stimulated the in vivo generation and expansion of melanoma antigen-reactive immune effectors that efficiently lysed the syngeneic melanoma cells; accordingly, the mice of these two groups developed smaller tumors.

### 2.4. In Vivo Administration of proTα/AWE or proTα(100–109)/AWE Enhanced Production of Th1-Polarizing Cytokines

**Figure 5:**
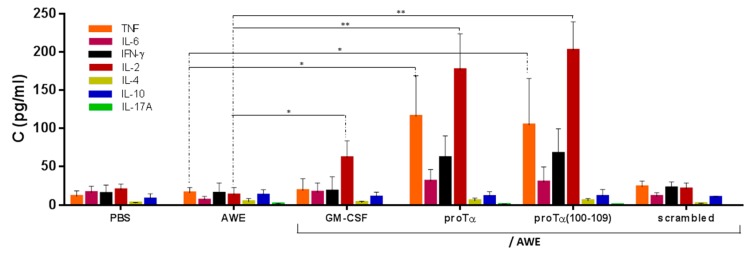
Proinflammatory and Th1-type cytokine levels were higher in serum of mice treated in vivo with proTα/AWE and proTα(100–109)/AWE. Mouse sera collected on day 34 were analyzed by flow cytometry using Cytometric Bead Array (CBA). Mean concentration of each cytokine (in pg/mL) ± SD from five mice/group is shown. * *p* < 0.05 and ** *p* < 0.01 compared to AWE.

Considering that effector immune cells are activated in the presence of stimulating cytokines, we further assessed the concentration of a panel of relevant cytokines in the serum of treated mice. In parallel to spleen cell isolation, serum from the same animals was collected on day 34 and analyzed with a Cytometric Bead Array (CBA), an assay that requires small amounts of serum and allows the concomitant measurement of multiple cytokines in the same sample. Thus, complete profiles for TNF, IL-6 (proinflammatory), IL-2, IFN-γ (Th1), IL-4, IL-10 (Th2), and IL-17A (Th17) from a single sample in a single experiment were compared between the groups of differentially treated mice. Marginal levels of all cytokines were detected in the sera of control animals, animals treated with AWE, scrambled peptide/AWE, and GM-CSF/AWE, with the exception of IL-2, which was increased to 61.8 pg/mL in the latter group (*p* < 0.05 compared to AWE; Figure 5). In mice treated with proTα/AWE or proTα(100–109)/AWE, we noticed a significant increase in serum concentration of proinflammatory and Th1-type cytokines (116.0 and 104.7 pg/mL for TNF; 31.2 and 30.1 pg/mL for IL-6; 176.9 and 202.4 pg/mL for IL-2; 62.0 and 67.5 pg/mL for IFN-γ, respectively; *p* < 0.05 for TNF and *p* < 0.01 for IL-2 compared to AWE), whereas low levels (< 20.0 pg/mL) were recorded for IL-4, IL-10, and IL-17A; as evident, the most significant induction referred to TNF and IL-2. These results suggest that the in vivo Th1-polarizing cytokine profile in the serum of mice with smaller melanoma tumor masses (i.e., proTα/AWE- and proTα(100–109)/AWE-treated) provided a supportive milieu for the expansion of antitumor-reactive effectors, mainly cytotoxic T and NK cells, as shown in Figure 4, which efficiently targeted and controlled melanoma-cell proliferation.

This was also verified at the end of the monitoring period (days 48–54), where sera from mice treated with proTα/AWE or proTα(100–109)/AWE had high concentrations of IFN-γ and low concentrations of IL-4, as determined by cytokine-specific ELISAs (Figure S3A); this was further confirmed using a 20-plex Luminex assay, where levels of Th1-associated proinflammatory cytokines (IL-1α, IL-1β, TNF-α, IL-6, IL-12) and chemokines (MCP-1, MIP-1α), as well as Th1-type cytokines (IL-2, IFN-γ), were higher in the serum of proTα/AWE- or proTα(100–109)/AWE-treated animals compared to other used combinatorial interventions (Figure S3B).

## 3. Discussion

The preclinical and subsequently clinical use of peptide-based anticancer vaccines is among the most widely exploited immunotherapeutics, aiming to stimulate cytotoxic effectors to target one or multiple tumor-associated antigens. Although peptide-based vaccines tested in clinical trials are usually well-tolerated and adequately immunogenic, their therapeutic value as standalone therapy has so far been limited [20]. A strategy explored to increase anticancer vaccine efficiency is the use of more potent immunostimulatory adjuvants [21]. In contrast to alum, which is a poor inducer of Th1-associated immune responses [22], Toll-like receptor (TLR) agonists, i.e., pathogen-associated molecular patterns (PAMPs), and danger-associated molecular patterns (DAMPs), shape the adaptive arm of immunity by recruiting immune cells such as DCs, CD8+ T and NK cells into the tumor microenvironment and bias the immune response towards Th1-type, further activating highly effective cytotoxic effectors [23]. ProTα belongs to the wide family of DAMPs [5,7,24] and has been reportedly shown by us and others to signal through TLR-4 [7,15,25,26,27,28]. Moreover, we identified the C-terminal decapeptide proTα(100–109) as the fragment responsible for the immunomodulating activity of the parent molecule [13,14,15]. In support of this, Wang et al. [29] showed that depriving proTα of its NLS (proTαΔNLS) attenuates the proinflammatory and consequently immunostimulating activity of the molecule.

In our previous in vitro studies, we showed that proTα and proTα(100–109) promote the phenotypic maturation of human monocyte-derived DCs [13], and these proTα- or proTα(100–109)-matured DCs are functionally competent, as they induce strong T-cell responses in the presence of HER-2/neu antigenic epitopes (CD8+ cytotoxic effectors and CD4+ HER-2/neu-reactive helper cells) [15]. We also showed that the adoptive transfer of ovarian-cancer ascites-derived lymphocytes, preactivated in vitro with proTα or proTα(100–109), effectively retarded tumor growth in SCID mice inoculated with autologous tumor cells and conferred a survival benefit to the animals [30].

Herein, we administered both molecules in mice with established subcutaneous (s.c.) melanoma tumors and confirmed, for the first time in vivo, their potent adjuvanticity, as in combination with autologous melanoma antigens, proTα and proTα(100–109) favored the expansion of antitumor-reactive effectors, and accordingly hindered melanoma tumor growth. The rationale of the designed protocol (Figure 1) was based on the following: (1) mice were treated therapeutically upon palpable tumor formation (day 15) to better simulate human disease; (2) each treatment cycle (prime-boost; two cycles in total) comprised two intraperitoneal (i.p.) injections of proTα or proTα(100–109) to mature and potently activate peritoneal macrophages followed by an i.p. injection of the vaccine, i.e., the B16.F1 extract (AWE) [31], as source of melanoma antigens to be captured and processed by activated macrophages. Similar protocols have also been used in cancer patients, where adjuvants (usually GM-CSF) were given intradermally prior to the peptide vaccine to recruit and preactivate antigen-presenting cells (APCs) [32,33,34]; (3) AWE was premixed with incomplete Freund’s adjuvant (IFA) to form a depot since IFA can be used in combination with other adjuvants without obstructing desirable Th1-type anticancer responses [35]; (4) GM-CSF was used as a control IMD due to its long-term use as standard adjuvant both in animals and in cancer patients [32,36]; (5) a scrambled peptide with the same amino acid composition as proTα(100–109) but a disrupted sequence served as control peptide to verify that the observed in vivo activity of the decapeptide is sequence-specific [13,14,15,16]; (6) proTα was administered at a total dose of ~0.1 mg/kg/mouse, analogous to doses reported in the literature [17,18,19,24,37], whereas the dose of proTα(100–109) was determined by dose-kinetic experiments (data not shown). The concomitant study of proTα(100–109) along with the native polypeptide is important since the former may eventually substitute for proTα due to its easier production at low cost and under good manufacturing practice (GMP) conditions; and (7) we additionally assessed the cytokine profile of mouse sera at the end of the follow-up period (days 48–54) to confirm that the adaptive Th1-biased immunity generated by proTα and proTα(100–109) was preserved even after end of treatment. 

Our results showed that mice treated with two cycles of IMD–IMD–peptide vaccine grew smaller tumors over time, and the difference in melanoma masses was statistically significant compared to the groups receiving the peptide vaccine (AWE) alone or with an inactive IMD (scrambled peptide/AWE). Noticeably, in contrast to proTα/AWE and proTα(100–109)/AWE, GM-CSF/AWE likely did not provide a sufficiently strong effector response that could lead to survival benefit (Figure 2). Although GM-CSF is considered the gold standard of peptide-based vaccination trials, reports have long shown that this growth factor per se does not always activate robust cytotoxic T lymphocyte (CTL) responses [38]. In addition, GM-CSF treatment typically induces a Th2-type humoral response rather than Th1 anticancer immunity [39], and its effect is dose-dependent [40]. Coadministration of GM-SCF and TLR agonists has also generated contradictory results, as certain TLR ligands, e.g., CpG or poly I:C, improve GM-CSF performance [38], while others like imiquimod negate its stimulatory effects [41]. Whether proTα or proTα(100–109), in synergy with GM-CSF or combined with one or more TLR agonists [42,43] or other immunoregulatory strategies (e.g., checkpoint blockade) [44,45], could generate higher levels of tumor-specific Th1 cells with active immunization, remains to be tested.

The in vivo antimelanoma activity of proTα and proTα(100–109) was comparable to the efficacy of other TLR agonists tested in similar preclinical models. For example, mice bearing B16.F10 melanomas administered poly-I:C (a TLR-3 agonist also known as Hiltonol) showed a ca. 65% decrease in tumor size [46]; in B16BL6-implanted mice, i.p. administration of CpG oligonucleotides (TLR-9 agonists) resulted in significantly lower (>50%) metastatic melanoma nodules in the lung [47], whereas CpG immunoconjugates (e.g., with a chimeric tumor necrosis therapy antibody) injected to B16-bearing mice performed better and resulted in ca. 60% melanoma tumor regression [48]; treating B16-inoculated mice with stress protein grp170 (likely a TLR-2/4 ligand) coupled to melanoma antigens augmented immune activation and showed potent therapeutic efficacy against the established melanomas, which regressed by ca. 90% [49]; finally, an immunoactive nonapeptide deriving from the prototype DAMP HMGB1, namely, UC1018 (possible ligand of TLR-4 and -2), was shown to protect mice from a highly lethal B16 challenge, whereas, impressively, 60% of the animals were shown to remain tumorfree [50]. In our melanoma model, two treatment cycles with proTα or proTα(100–109) led to a ca. 85% reduction in tumor growth compared to the respective control (AWE group), as estimated by the size of melanoma tumors recorded on day 34.

To mechanistically explain the observed antimelanoma effect of proTα and proTα(100–109) in vivo, we studied three parameters that contribute to the inhibition of tumor growth: (1) histological characteristics, architecture, and lymphocyte infiltration of the tumor; (2) cytotoxicity of total spleen cells that comprise both T- and NK cells; and (3) cytokine profile in the serum of treated mice. We found that melanomas from mice administered proTα or proTα(100–109) and the vaccine were not only smaller, but had fewer necrotic lesions and were enriched in T cells, indicating a selective elimination of melanoma cells, also evident by less melanin production and reduced tumor-cell invasiveness (Figure 3). Similarly to melanoma patients, where high levels of tumor-infiltrating lymphocytes associate with improved overall survival [51], the mice of the aforementioned groups exhibited slower tumor progression and survived longer. Moreover, total spleen cells isolated from mice treated with proTα/AWE and proTα(100–109)/AWE efficiently killed autologous B16.F1- and NK/LAK-sensitive targets, a result verifying the in vivo generation of functionally competent antimelanoma-reactive T and NK effectors. This finding is in agreement with a series of reports showing that proTα and proTα(100–109) enhance the in vitro and in vivo cytotoxicity of both MHC- and non-MHC-restricted effector cells (T and NK/LAK cells, respectively) and this effect is IL-2-dependent (reviewed in [5,52]). Thus, we next assessed whether melanoma T-cell invasion and cytotoxic responses were sustained by a favorable cytokine profile, in this case, of the Th1-type. Indeed, 10 days after the end of cycle 2, we detected much higher concentrations (4.5- to 7.2-fold compared to the AWE group) of proinflammatory (TNF, IL-6) and Th1-skewing (IFN-γ, IL-2) cytokines in the serum of mice treated with proTα/AWE or proTα(100–109)/AWE. Surprisingly, much lower levels of all cytokines were detected in the serum of GM-CSF/AWE-treated animals. What was even more interesting was that the Th1 high/Th2 low balance was evident until the end of the monitoring period (i.e., ca. one month after the last injection when all mice were euthanized), suggesting that proTα and proTα(100–109) also effectively primed in vivo the activation of APCs and CD4+ Th1-helper effectors. The cytokine milieu produced in vivo in response to proTα and proTα(100–109) stimulation, was documented in various mouse and human experiment settings [12,15,17,19,53,54]. Notably, the group of Arevik Mosoian [25] elegantly showed for the first time that proTα and fragments thereof stimulate the production of RANTES, a chemokine known to recruit tumor-reactive immune cells into the tumor. In agreement with this, MIP-1α and MCP-1, which act similarly to RANTES [55], were found elevated in the serum of proTα- and proTα(100–109)-treated mice, further supporting our immunohistochemistry results and the therapeutic advantage observed in these groups.

## 4. Materials and Methods

### 4.1. Proteins and Peptides

Recombinant mouse GM-CSF was purchased from R & D Systems GmbH (Wiesbaden-Nordenstadt, Germany). Human recombinant proTα was obtained from Alexis Biochemicals (San Diego, CA, USA) and passed through an endotoxin removal column (Pierce Biotechnology Inc., Waltham, IL, USA) prior to experiment use. Decapeptide proTα(100–109) (TKKQKTDEDD) and the scrambled decapeptide (KETDKDKTDQ) were synthesized as previously reported [15] following Fmoc/tBu chemistry on a multiple peptide synthesizer Syro II (MultiSynTech, Witten, Germany), purified using RP-HPLC (purity exceeded 95%), and characterized using ESI-MS. Prior to their use, all proteins and peptides were tested for endotoxin levels using the LAL Chromogenic Endotoxin Quantitation Kit (Pierce Biotechnology); endotoxin levels in all cases were below the detection limit of the Kit, i.e., <0.1 endotoxin units/mL.

### 4.2. Cell Cultures

The murine cell lines B16.F1 (melanoma), YAC-1 (lymphoma), and P815 (mastocytoma) were obtained from the European Collection of Authenticated Cell Cultures (ECACC, Salisbury, UK). Cell lines and mouse total spleen cells were maintained in RPMI-1640, supplemented with 10% heat-inactivated fetal bovine serum, 2 mM L-glutamine, 10 mM HEPES, 5 μg/mL gentamycin, 10 U/mL penicillin, 10 U/mL streptomycin (all from Lonza, Cologne, Germany), and 50 μM β-mercaptoethanol (Sigma-Aldrich, Darmstadt, Germany; thereafter referred to as complete medium) at 37 °C in a humidified 5% CO_2_ incubator.

### 4.3. Preparation of AWE

Melanoma-associated antigenic peptides were extracted from the surface of B16.F1 cells (AWE) as previously described [31,56] with minor modifications (Figure S1). In brief, for each vaccine dose, 2 × 10^7^ B16.F1 were washed with HBSS, followed by a short (5 min) incubation in citrate buffer (0.131 M citric acid; 0.066 M Na_2_HPO_4_; 0.15 M NaCl), pH 3.0. Acid-eluted peptides were clarified by 2 successive centrifugations and immediately processed on a SepPak C18 cartridge (Waters, Millipore, Milford, MA, USA). The peptide-bound material was eluted from the column with 5 mL 80% (v/v) acetonitrile and lyophilized. The dry product was reconstituted in HBSS and passed through a 10,000 Da molecular weight cut-off filter (Centricon centrifuge concentrator, Amicon, Beverly, MA, USA). The final filtrate was aliquoted and stored at −20 °C.

### 4.4. Establishment of In Vivo Melanoma Mouse Model

Male C57BL/6 mice (6–8 weeks; 15–20 g) were purchased from the Hellenic Pasteur Institute. All animals were housed under standard laboratory conditions of temperature (22 °C) and light (photoperiod 12:12 h light:dark) with free access to food and water. Experiments were performed in accordance with Law 2015/27.2.1992, Presidential Decree 160/3.5.1991, and Directive 86/609/EEC/ 24.11.1986 of the Council of Europe on Animal Welfare. The study was approved by the Ethics and Biosafety Committee of the Hellenic Pasteur Institute (ethical protocol code K9556, 2 July 2008).

B16.F1 melanoma cells (syngeneic to C57BL/6 mice) were expanded to sufficient numbers in complete medium. On day 0, mice were s.c. inoculated with B16.F1 (10^5^ cells/mouse in 200 μL PBS). The number of inoculated cells was selected on the basis of preliminary in vivo titration studies (Figure S2), considering the optimal balance between rate of tumor growth and the duration of the protocol. On day 15, when tumors were palpable, animals were randomly assigned to 6 groups (8–10 mice/group; Figure 1). The therapeutic vaccination protocol was as follows: on days 15 and 17, mice were i.p. injected with PBS (group A; control) or the IMDs GM-CSF (100 ng/mouse), proTα (350 ng/mouse), proTα(100–109) (200 ng/mouse), or scrambled decapeptide (200 ng/mouse), all diluted in 0.5 mL PBS. On day 18, all groups except for the control were i.p. vaccinated with a 1:1 mixture of AWE (100 μL AWE, comprising peptides eluted from 2 × 10^7^ B16.F1) and IFA (100 μL IFA). This IMDs-IMDs-AWE administration cycle was repeated as such once more after 1 week (i.e., on days 21, 23, and 24).

Tumor growth was recorded every 2 to 3 days by measuring major and minor axes of the s.c. formed masses via a digital caliper. Measurements were transformed into tumor volume using the formula: tumor volume (cm^3^) = major axis × minor axis^2^ × 0.5. On day 34, when the mean tumor volume of group A reached ~2.5 cm^3^, 5 animals from each group were euthanized by cervical dislocation. Tumors were excised and snap-frozen in liquid nitrogen. Spleens were aseptically excised, individually homogenized, and total spleen cells were tested for their cytotoxicity versus B16.F1, YAC-1, and P815 targets. Serum was collected by centrifuging mouse blood at 2500 g for 15 min on day 34 (Sampling 1), and on days 48 (groups B, C, F) and 54 (groups D, E) (Sampling 2).

### 4.5. Histological and Immunohistochemical Analyses of Tumor Sections

Formalin-fixed and paraffin-embedded tumor sections were stained with hematoxylin-eosin (H and E; Sigma-Aldrich) for standard histological analysis, and sections were independently evaluated by two blinded pathologists. Tissue-infiltrating T cells were determined by immunohistochemistry using a rabbit polyclonal antibody to CD3 (Abcam, Cambridge, UK; #ab5690; diluted 1:200 in 1×TBS). Sections were stained using an Ultravision LP Detection System Kit (Thermo Scientific, Waltham, MA, USA), and color development was achieved by 3,3′-diaminobenzidine tetrahydrochloride (DAB) and hematoxylin as counterstain. Slides were observed under a light microscope (Leica DM LB2, Richmond Scientific, Chorley, UK) and photographs were taken using a Leica DFC 320 camera (Richmond Scientific). The evaluation of CD3+ T cells was performed by counting the number of positive immune cells per high power field (magnification ×400).

### 4.6. Cytotoxicity Assay

The ex vivo cytolytic capacity of T cells was evaluated by standard ^51^Cr-release assay, as previously described [57]. For this, freshly isolated murine total spleen cells were used as effectors (E) versus intracellularly ^51^Cr-labelled B16.F1, YAC-1 (NK-sensitive), and P815 (LAK-sensitive) tumor cell targets (T) at E:T ratios of 25:1, 50:1, and 100:1. Only data at the 100:1 ratio are shown, as differences in % cytotoxicity between groups at 25:1 and 50:1 ratios were very low and thus could not be safely evaluated. In all experiments, effectors and targets were coincubated for 4–6 h.

### 4.7. Measurement of Cytokine Levels

Cytokine analysis was conducted using the BD™ CBA Mouse Th1/Th2/Th17 Kit (BD Biosciences, San Jose, CA, USA) that allows for the simultaneous detection of TNF, IL-6, IFN-γ, IL-2, IL-4, IL-10, and IL-17A. Serum samples were thawed and diluted with assay diluent (1:2 v/v), and CBA analysis was performed in accordance with the instructions of the Kit. Briefly, 50 μL of the capture bead mixture was added to 50 μL of serum, followed by the addition of 50 μL of phycoerythrin-conjugated detection antibody, and the final mixture was incubated for 3 h in the dark with occasional shaking. Samples were washed with 1 mL wash buffer (200× *g*; 5 min), and the pellet was resuspended in 300 μL wash buffer. Two hundred μL of each sample was analyzed on a FACSCanto II (BD Biosciences). Prior to data acquisition, the instrument was checked for sensitivity and overall performance using Cytometer Setup and Tracking (CS and T) beads (BD Biosciences). Results were calculated with the FCAP Array Software (v1.1.1., Soft Flow Hungary Ltd, Pécs, Hungary). 

Commercially available ELISA Kits, specific for mouse IFN-γ and IL-4, were purchased from Thermo Fisher Scientific and performed following manufacturer instructions. Serum samples were also analyzed using a mouse cytokine 20-plex Luminex Kit (Thermo Fisher Scientific) as per manufacturer instructions (details in Figure S3).

### 4.8. Statistical Analysis

All in vivo and ex vivo experiments were conducted at least 3 times. Data were analyzed using GraphPad Prism 7 software (San Diego, CA, USA). Results are expressed as means ± standard deviation (SD). For statistical analysis, the Student’s t-test was used. *p*-Values < 0.05 were considered statistically significant. Tumor size among groups was compared with Wilcoxon’s signed-rank test.

## 5. Conclusions

**Figure 6:**
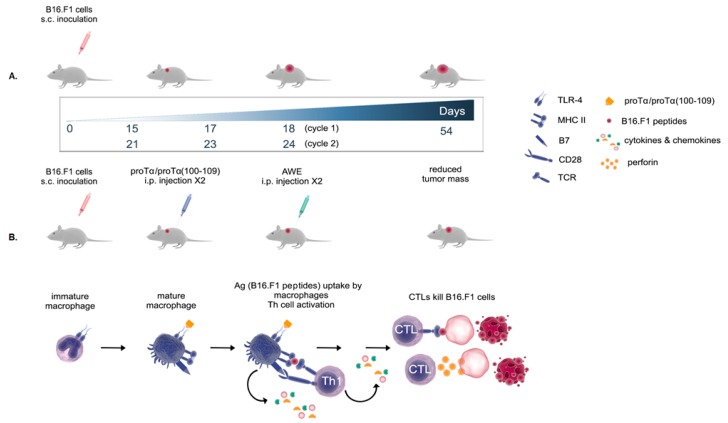
Proposed scenario of in vivo mechanism of action of proTα and proTα(100–109) as adjuvants. Intraperitoneally administered proTα or proTα(100–109) in melanoma-bearing mice bind to and signal via TLR-4 (possibly more pattern-recognition receptors (PRRs), not shown) on peritoneal macrophages (or other TLR-expressing APCs, not shown) leading to their activation. Macrophages acquire a mature phenotype and efficiently uptake vaccine melanoma antigens (AWE), while at the same time overexpress MHC class I (not shown) and II molecules, and costimulatory signals, and secrete proinflammatory cytokines and chemokines. Immune synapses formed between melanoma antigens presented on MHC class I and II molecules, and cytotoxic and helper T cells, lead to their differentiation to highly lytic CTLs and Th1-type effectors, respectively. The latter secrete Th1-polarizing cytokines (see Figure 5) further supporting the cytolytic program of CTLs (and likely of colocalized NK cells, not shown). CTLs migrate through the lymphoid system, infiltrate tumors, and exert their melanoma-reactive cytotoxicity in situ. As a result, mice develop smaller melanoma tumors over time (**B**). In mice receiving no treatment (**A**), melanoma cells rapidly proliferate, generating larger tumor masses and reducing animal lifespan.

In conclusion, for the first time in vivo, we showed that DAMPs proTα and proTα(100–109) act as adjuvants and improve the immunogenicity of peptide-based anticancer vaccines. The mechanism of action underlying the observed melanoma-growth inhibition involves the activation of TLR-4 on APCs (peritoneal macrophages herein) by proTα and proTα(100–109), their subsequent maturation, uptake and presentation of melanoma antigens (AWE) to T cells, a process accompanied by the secretion of proinflammatory cytokines and chemokines. Effector helper T cells secrete Th1-polarizing cytokines and stimulate melanoma-reactive cytotoxic T cells and NK cells that are consequently recruited to the tumor site and inhibit melanoma-cell proliferation (Figure 6).

Although our therapeutic vaccination protocol controlled melanoma growth, no complete tumor regression was observed, and all mice eventually succumbed to the disease. Administration of more booster cycles is probably needed to allow sufficient time for mounting a stronger immune response leading to malignant-cell elimination. Nevertheless, we provide evidence that peptide-based vaccines injected in combination with proTα or proTα(100–109), which provide strong danger/activation signals to the innate immune system, may hold promise in the clinic and could complement currently applied anticancer-vaccine strategies.

## Supplementary Materials

The following are available online at https://www.mdpi.com/2072-6694/11/11/1764/s1: Figure S1: Schematic presentation of melanoma-antigen extract preparation (acid wash extract; AWE) used as tumor-associated peptide vaccine, Figure S2: In vivo titration studies of B16.F1 cells injected s.c. to C57BL/6 mice, Figure S3: Assessment of cytokine and chemokine levels in mice serum.

## References

[1] Haritos A.A., Goodall G.J., Horecker B.L. (1984). Prothymosin alpha: Isolation and properties of the major immunoreactive form of thymosin alpha 1 in rat thymus. Proc. Natl. Acad. Sci. USA.

[2] Gómez-Márquez J., Rodríguez P. (1998). Prothymosin α is a chromatin-remodelling protein in mammalian cells. Biochem. J..

[3] Eschenfeldt W.H., Berger S.L. (1986). The human prothymosin alpha gene is polymorphic and induced upon growth stimulation: Evidence using a cloned cDNA. Proc. Natl. Acad. Sci. USA.

[4] Jiang X., Kim H.E., Shu H., Zhao Y., Zhang H., Kofron J., Donnelly J., Burns D., Ng S.C., Rosenberg S. (2003). Distinctive roles of PHAP proteins and prothymosin-alpha in a death regulatory pathway. Science.

[5] Samara P., Karachaliou C.E., Ioannou K., Papaioannou N.E., Voutsas I.F., Zikos C., Pirmettis I., Papadopoulos M., Kalbacher H., Livaniou E. (2017). Prothymosin alpha: An alarmin and more…. Curr. Med. Chem..

[6] Salvin S.B., Horecker B.L., Pan L.X., Rabin B.S. (1987). The effect of dietary zinc and prothymosin alpha on cellular immune responses of RF/J mice. Clin. Immunol. Immunopathol..

[7] Mosoian A., Teixeira A., Burns C.S., Sander L.E., Gusella G.L., He C., Blander J.M., Klotman P., Klotman M.E. (2010). Prothymosin-alpha inhibits HIV-1 via Toll-like receptor 4-mediated type I interferon induction. Proc. Natl. Acad. Sci. USA.

[8] Baxevanis C.N., Sfagos C., Anastasopoulos E., Reclos G.J., Papamichail M. (1990). Prothymosin-alpha enhances HLA-DR antigen expression on monocytes from patients with multiple sclerosis. J. Neuroimmunol..

[9] Baxevanis C.N., Thanos D., Reclos G.J., Anastasopoulos E., Tsokos G.C., Papamatheakis J., Papamichail M. (1992). Prothymosin alpha enhances human and murine MHC class II surface antigen expression and messenger RNA accumulation. J. Immunol..

[10] Skopeliti M., Kratzer U., Altenberend F., Panayotou G., Kalbacher H., Stevanovic S., Voelter W., Tsitsilonis O.E. (2007). Proteomic exploitation on prothymosin α-induced mononuclear cell activation. Proteomics.

[11] Baxevanis C.N., Frillingos S., Seferiadis K., Reclos G.J., Arsenis P., Katsiyiannis A., Anastasopoulos E., Tsolas O., Papamichail M. (1990). Enhancement of human T lymphocyte function by prothymosin α: Increased production of interleukin-2 and expression of interleukin-2 receptors in normal human peripheral blood T lymphocytes. Immunopharmacol. Immunotoxicol..

[12] Cordero O.J., Sarandeses C.S., López J.L., Cancio E., Regueiro B.J., Nogueira M. (1991). Prothymosin α enhances interleukin 2 receptor expression in normal human T-lymphocytes. Int. J. Immunopharmacol..

[13] Skopeliti M., Iconomidou V.A., Derhovanessian E., Pawelec G., Voelter W., Kalbacher H., Hamodrakas S.J., Tsitsilonis O.E. (2009). Prothymosin α immunoactive carboxyl-terminal peptide TKKQKTDEDD stimulates lymphocyte reactions, induces dendritic cell maturation and adopts a β-sheet conformation in a sequence-specific manner. Mol. Immunol..

[14] Samara P., Ioannou K., Neagu M., Arnogiannaki N., Ardavanis A., Voelter W., Tsitsilonis O. (2013). The C-terminal decapeptide of prothymosin α is responsible for its stimulatory effect on the functions of human neutrophils in vitro. Int. Immunopharmacol..

[15] Ioannou K., Derhovanessian E., Tsakiri E., Samara P., Kalbacher H., Voelter W., Trougakos I.P., Pawelec G., Tsitsilonis O.E. (2013). Prothymosin α and a prothymosin α-derived peptide enhance T_H_1-type immune responses against defined HER-2/neu epitopes. BMC Immunol..

[16] Karachaliou C.E., Triantis C., Liolios C., Palamaris L., Zikos H., Tsitsilonis O.E., Kalbacher H., Voelter W., Loudos G., Papadopoulos M. (2017). In vivo biodistribution and imaging studies with a ^99m^Tc-radiolabeled derivative of the C-terminus of prothymosin alpha in mice bearing experimentally-induced inflammation. Eur. J. Pharm. Biopharm..

[17] Papanastasiou M., Baxevanis C.N., Papamichail M. (1992). Promotion of murine antitumor activity by prothymosin alpha treatment: I. Induction of tumoricidal peritoneal cells producing high levels of tumour necrosis factor alpha. Cancer Immunol. Immunother..

[18] Baxevanis C.N., Gritzapis A.D., Dedoussis G.V., Papadopoulos N.G., Tsolas O., Papamichail M. (1994). Induction of lymphokine-activated killer activity in mice by prothymosin α. Cancer Immunol. Immunother..

[19] Baxevanis C.N., Gritzapis A.D., Spanakos G., Tsitsilonis O.E., Papamichail M. (1995). Induction of tumor-specific T lymphocyte responses in vivo by prothymosin α. Cancer Immunol. Immunother..

[20] Thomas S., Pendergast G.C. (2016). Cancer vaccines: A brief overview. Methods Mol. Biol..

[21] Bezu L., Kepp O., Cerrato G., Pol J., Fucikova J., Spisek R., Zitvogel L., Kroemer G., Galluzzi L. (2018). Trial watch: Peptide-based vaccines in anticancer therapy. Oncoimmunology.

[22] Mbow M.L., De Gregorio E., Valiante N.M., Rappuoli R. (2010). New adjuvants for human vaccines. Curr. Opin. Immunol..

[23] Baxevanis C.N., Voutsas I.F., Tsitsilonis O.E. (2013). Toll-like receptor agonists: Current status and future perspective on their utility as adjuvants in improving anticancer vaccination strategies. Immunotherapy.

[24] Maeda S., Sasaki K., Halder S.K., Fujita W., Ueda H. (2016). Neuroprotective DAMPs member prothymosin alpha has additional beneficial actions against cerebral ischemia-induced vascular damages. J. Pharmacol. Sci..

[25] Gusella G.L., Teixeira A., Aberg J., Uversky V.N., Mosoian A. (2016). Prothymosin-α variants elicit anti-HIV-1 response via TLR4 dependent and independent pathways. PLoS ONE.

[26] Halder S.K., Matsunaga H., Ishii K.J., Ueda H. (2015). Prothymosin-alpha preconditioning activates TLR4-TRIF signaling to induce protection of ischemic retina. J. Neurochem..

[27] Omotuyi O., Matsunaga H., Ueda H. (2015). Evidence for proTα-TLR4/MD-2 binding: Molecular dynamics and gravimetric assay studies. Expert Opin. Biol. Ther..

[28] Cordero O.J. (2011). Data on the interaction between prothymosin α and TLR4 may help to the design of new antiviral compounds. J. Acquir. Immune Defic. Syndr..

[29] Wang L.C., Wu C.L., Cheng Y.Y., Tsai K.J. (2017). Deletion of nuclear localizing signal attenuates proinflammatory activity of prothymosin-alpha and enhances its neuroprotective effect on transient ischemic stroke. Mol. Neurobiol..

[30] Voutsas I.F., Pistamaltzian N., Tsiatas M.L., Skopeliti M., Katsila T., Mavrothalassiti I., Spyrou S., Dimopoulos M.A., Tsitsilonis O.E., Bamias A. (2013). Ovarian malignant ascites-derived lymphocytes stimulated with prothymosin α or its immunoactive decapeptide lyse autologous tumour cells in vitro and retard tumour growth in SCID mice. Eur. J. Cancer.

[31] Nair S.K., Boczkowski D., Snyder D., Gilboa E. (1997). Antigen-presenting cells pulsed with unfractionated tumor-derived peptides are potent tumor vaccines. Eur. J. Immunol..

[32] Gjertsen M.K., Buanes T., Rosseland A.R., Bakka A., Gladhaug I., Søreide O., Eriksen J.A., Møller M., Baksaas I., Lothe R.A. (2001). Intradermal ras peptide vaccination with granulocyte-macrophage colony-stimulating factor as adjuvant: Clinical and immunological responses in patients with pancreatic adenocarcinoma. Int. J. Cancer.

[33] Rahma O.E., Herrin V.E., Ibrahim R.A., Toubaji A., Bernstein S., Dakheel O., Steinberg S.M., Abu Eid R., Mkrtichyan M., Berzofsky J.A. (2014). Pre-immature dendritic cells (PIDC) pulsed with HPV16 E6 or E7 peptide are capable of eliciting specific immune response in patients with advanced cervical cancer. J. Transl. Med..

[34] Nitschke N.J., Bjoern J., Iversen T.Z., Andersen M.H., Svane I.M. (2017). Indoleamine 2,3-dioxygenase and survivin peptide vaccine combined with temozolomide in metastatic melanoma. Stem Cell Investig..

[35] Jensen F.C., Savary J.R., Diveley J.P., Chang J.C. (1998). Adjuvant activity of incomplete Freund’s adjuvant. Adv. Drug Deliv. Rev..

[36] Yan W.L., Shen K.Y., Tien C.Y., Chen Y.A., Liu S.J. (2017). Recent progress in GM-CSF-based cancer immunotherapy. Immunotherapy.

[37] Halder S.K., Sugimoto J., Matsunaga H., Ueda H. (2013). Therapeutic benefits of 9-amino acid peptide derived from prothymosin alpha against ischemic damages. Peptides.

[38] Ali O.A., Verbeke C., Johnson C., Sands R.W., Lewin S.A., White D., Doherty E., Dranoff G., Mooney D.J. (2014). Identification of immune factors regulating antitumor immunity using polymeric vaccines with multiple adjuvants. Cancer Res..

[39] He L.Z., Weidlick J., Sisson C., Marsh H.C., Keler T. (2015). Toll-like receptor agonists shape the immune responses to a mannose receptor-targeted cancer vaccine. Cell. Mol. Immunol..

[40] Qiu J.T., Alson D., Lee T.H., Tsai C.C., Yu T.W., Chen Y.S., Cheng Y.F., Lin C.C., Schuyler S.C. (2019). Effect of multiple vaccinations with tumor cell-based vaccine with codon-modified GM-CSF on tumor growth in a mouse model. Cancers (Basel).

[41] Dang Y., Wagner W.M., Gad E., Rastetter L., Berger C.M., Holt G.E., Disis M.L. (2012). Dendritic cell-activating vaccine adjuvants differ in the ability to elicit antitumor immunity due to an adjuvant-specific induction of immunosuppressive cells. Clin. Cancer Res..

[42] Lövgren T., Sarhan D., Truxová I., Choudhary B., Maas R., Melief J., Nyström M., Edbäck U., Vermeij R., Scurti G. (2017). Enhanced stimulation of human tumor-specific T cells by dendritic cells matured in the presence of interferon-γ and multiple Toll-like receptor agonists. Cancer Immunol. Immunother..

[43] Wells J.W., Cowled C.J., Farzaneh F., Noble A. (2008). Combined triggering of dendritic cell receptors results in synergistic activation and potent cytotoxic immunity. J. Immunol..

[44] Davila E., Kennedy R., Celis E. (2003). Generation of antitumor immunity by cytotoxic T lymphocyte epitope peptide vaccination, CpG-oligodeoxynucleotide adjuvant, and CTLA-4 blockade. Cancer Res..

[45] Tian H., Shi G., Wang Q., Li Y., Yang Q., Li C., Yang G., Wu M., Xie Q., Zhang S. (2016). A novel cancer vaccine with the ability to simultaneously produce anti-PD-1 antibody and GM-CSF in cancer cells and enhance Th1-biased antitumor immunity. Signal Transduct. Target. Ther..

[46] Nagato T., Lee Y.-R., Harabuchi Y., Celis E. (2014). Combinatorial immunotherapy of polyinosinic-polycytidylic acid and blockade of programmed death-ligand 1 induce effective CD8 T-cell responses against established tumors. Clin. Cancer Res..

[47] Cho H.C., Kim B.H., Kim K., Park J.Y., Chang J.H., Kim S.K. (2008). Cancer immunotherapeutic effects of novel CpG ODN in murine tumor model. Int. Immunopharmacol..

[48] Jang J.K., Khawli L.A., Canter D.C., Hu P., Zhu T.H., Wu B.W., Angell T.E., Li Z., Epstein A.L. (2016). Systemic delivery of chTNT-3/CpG immunoconjugates for immunotherapy in murine solid tumor models. Cancer Immunol. Immunother..

[49] Wang X.Y., Sun X., Chen X., Facciponte J., Repasky E.A., Kane J., Subjeck J.R. (2010). Superior antitumor response induced by large stress protein chaperoned protein antigen compared with peptide antigen. J. Immunol..

[50] Saenz R., Messmer B., Futalan D., Tor Y., Larsson M., Daniels G., Esener S., Messmer D. (2014). Activity of the HMGB1-derived immunostimulatory peptide Hp91 resides in the helical C-terminal portion and is enhanced by dimerization. Mol. Immunol..

[51] Fu Q., Chen N., Ge C., Li R., Li Z., Zeng B., Li C., Wang Y., Xue Y., Song X. (2019). Prognostic value of tumor-infiltrating lymphocytes in melanoma: A systematic review and meta-analysis. Oncoimmunology.

[52] Samara P., Ioannou K., Tsitsilonis O.E. (2016). Prothymosin alpha and immune responses: Are we close to potential clinical applications?. Vitam. Horm..

[53] Eckert K., Grünberg E., Garbin F., Maurer H.R. (1997). Preclinical studies with prothumosin α1 on mononuclear cells from tumor patients. Int. J. Immunopharmacol..

[54] Cordero O.J., Sarandeses C., López-Rodriguez J.L., Nogueira M. (1995). The presence and cytotoxicity of CD16+ CD2- subset from PBL and NK cells in long-term IL-2 cultures enhanced by prothymosin-α. Immunopharmacology.

[55] Schaller T.H., Batich K.A., Suryadevara C.M., Desai R., Sampson J.H. (2017). Chemokines as adjuvants for immunotherapy: Implications for immune activation with CCL3. Expert Rev. Clin. Immunol..

[56] Baxevanis C.N., Voutsas I.F., Tsitsilonis O.E., Gritzapis A.D., Sotiriadou R., Papamichail M. (2000). Tumor-specific CD4+ T lymphocytes from cancer patients are required for optimal induction of cytotoxic T cells against the autologous tumor. J. Immunol..

[57] Ioannou K., Cheng K.F., Crichlow G.V., Birmpilis A.I., Lolis E.J., Tsitsilonis O.E., Al-Abed Y. (2014). ISO-66, a novel inhibitor of macrophage migration inhibitory factor, shows efficacy in melanoma and colon cancer models. Int. J. Oncol..

