# Supplementary material for: Antitumor Reactive T-Cell Responses Are Enhanced In Vivo by DAMP Prothymosin Alpha and Its C-Terminal Decapeptide
Source: Cancers (Basel). 2019 Nov 9;11(11):1764. doi: 10.3390/cancers11111764 (PMC6896021; doi:10.3390/cancers11111764)
Supplement: Supplementary file 1 [file cancers-11-01764-s001.pdf]

*Supplementary Materials*

# Antitumor Reactive T-Cell Responses Are Enhanced In Vivo by DAMP Prothymosin Alpha and Its C-Terminal Decapeptide

**Anastasios I. Birmpilis, Chrysoula-Evangelia Karachaliou, Pinelopi Samara, Kyriaki Ioannou, Platon Selemenakis, Ioannis V. Kostopoulos, Nadia Kavrochorianou, Hubert Kalbacher, Evangelia Livaniou, Sylva Haralambous, Athanasios Kotsinas, Farzin Farzaneh, Ioannis P. Trougakos, Wolfgang Voelter, Meletios-Athanasios Dimopoulos, Aristotelis Bamias and Ourania Tsitsilonis**

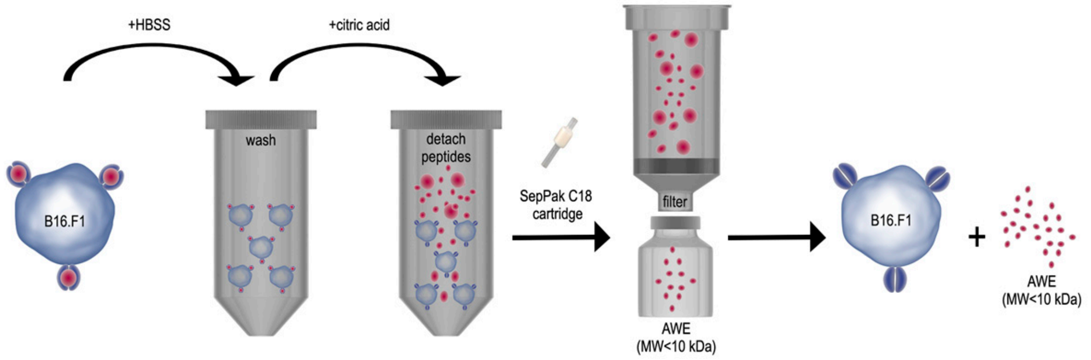


**Figure S1.** Schematic presentation of the melanoma antigen extract preparation (acid wash extract; AWE) used as tumor-associated peptide vaccine. B16.F1 cells were washed with HBSS and incubated for 5 min in citrate buffer pH 3.0. After centrifugation to remove cell debris, the supernatant containing the detached peptides was immediately processed on a pre-conditioned (with 10 mL ethanol and 20 mL d. $H_2O$) SepPak C18 cartridge. The peptide-bound material was eluted from the column with 5 mL 80% acetonitrile, lyophilized to dry and reconstituted in 1 mL HBSS. To recover peptides with MW < 10 kDa, the peptide mixture was filtered through a Centricon filter and the flow through material was used as peptide vaccine. Each dose being administered to mice contained peptides that were eluted from $2 \times 10^7$ B16.F1 cells.

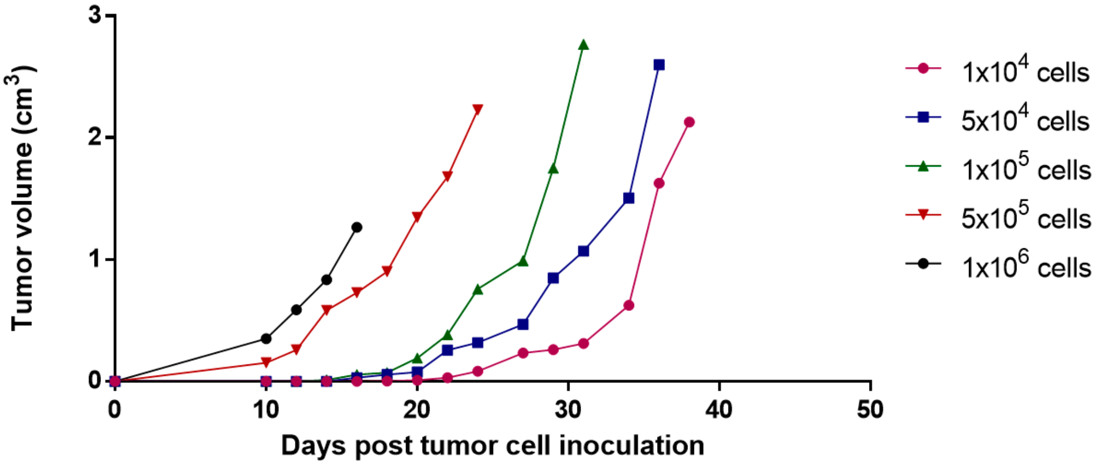


**Figure S2.** In vivo titration studies of B16.F1 cells injected s.c. to C57BL/6 mice. Tumor growth was recorded every 2 to 3 days by measuring the major and minor axes of the masses via a digital caliper. Tumor volumes were calculated using the formula: tumor volume ($cm^3$) = major axis × minor axis$^2$ × 0.5. Mean volumes from 4 animals/B16.F1 dose are shown. SDs are omitted for clarity. For the

establishment of the in vivo melanoma mouse model used herein, we selected to inject mice with 1 × $10^5$ melanoma cells, a dose showing the desirable tumor-growth rate profile.

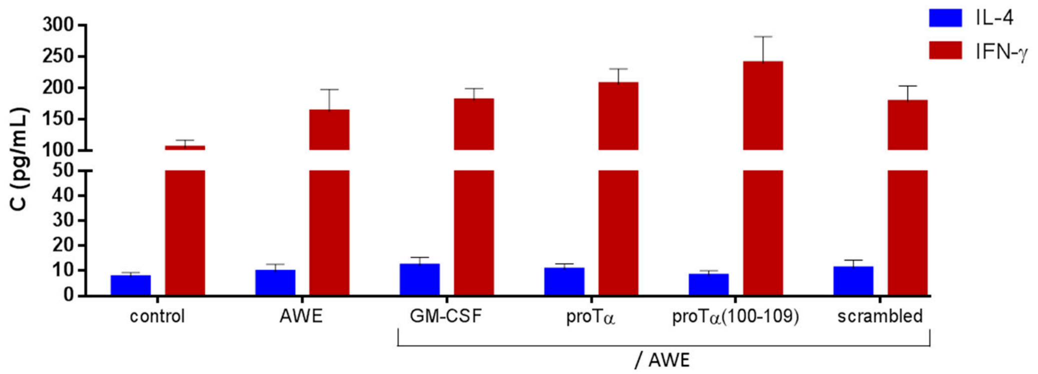


(A)

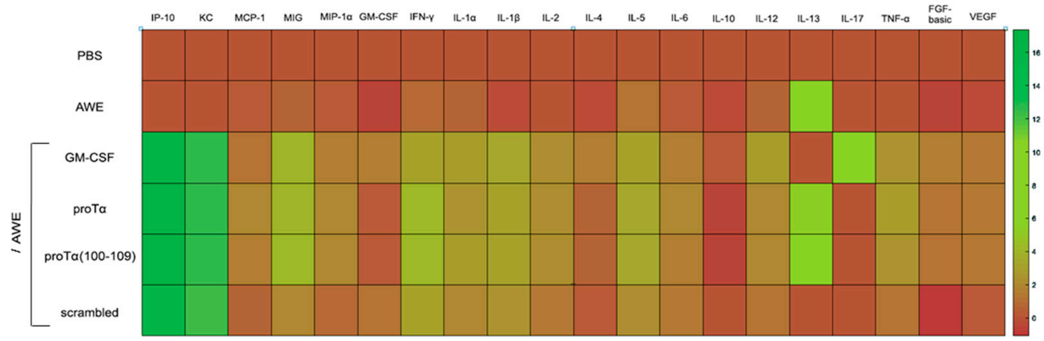


(B)

**Figure S3.** Assessment of cytokine and chemokine levels in mice serum. The sera used were collected: for the PBS group on day 34; for AWE, GM-CSF and scrambled groups on day 48; for proTα and proTα(100-109) groups on day 54. (**A**) Sera were assessed in duplicate with ELISAs specific for the cytokines IFN-γ (Th1) and IL-4 (Th2), performed according to the manufacturers' instructions. Mean concentrations ± SD from 4-5 animals are shown. Based on the levels of IFN-γ, the in vivo predominance of Th1-type immune responses seems likely. (**B**) The same sera were assessed with a 20-plex Luminex assay. Samples were diluted 1:2 in assay diluent; 25 μL of 1× antibody beads were added into each well of a 96-well flat bottom plate; wells were washed with 200 μL of 1× wash solution; 50 μL incubation buffer was added; 100 μL of diluted standards, blank and diluted samples were added in the relevant wells; the plate was covered and incubated on an orbital plate shaker (2 h; room temperature); wells were washed twice with 200 μL 1× wash solution; 1× biotinylated detector antibody (100 μL biotin diluent and 10 μL 10× biotinylated antibody/well) was added; the plate was incubated on an orbital shaker (1 h; room temperature); wells were washed twice with 200 μL 1× wash solution; 100 μL 1× streptavidin-RPE solution (100 μL RPE-diluent and 10 μL 10× streptavidin-RPE/well) was added; the plate was incubated on an orbital shaker (30 min; room temperature); wells were washed thrice with 200 μL 1× wash solution; 150 μL 1× wash solution was added to each well; the plate was placed on a shaker for 2 to 3 min and then analyzed into a Luminex® 100/200™ instrument. Cytokine and chemokine concentrations were determined from the standard curve using curve fitting software. Mean values from sera of 4–5 animals were calculated and expressed in a heatmap (MATLAB software, by MathWorks®) as fold-increase compared to the levels detected in the PBS-treated group defined as 0. The colors of the heatmap were mapped linearly as in the color key shown (low concentration in brown; high concentration in green).The levels of proinflammatory cytokines (IL-1α, IL-1β, TNF-α, IL-6, IL-12) and chemokines (MCP-1, MIP-1α) as well as Th1-type cytokines (IL-2, IFN-γ) were found increased in the serum of proTα/AWE- and proTα(100-109)/AWE-treated animals.

© 2019 by the authors. Licensee MDPI, Basel, Switzerland. This article is an open access article distributed under the terms and conditions of the Creative Commons Attribution (CC BY) license (http://creativecommons.org/licenses/by/4.0/).
